# Bibliography

**Published:** 1894-06

**Authors:** 


### Bibliography.

The Etiology of Osseous Deformities of the Head, Face, Jaws, and Teeth. By Eugene S. Talbot, M.D., D.D.S. Third Edition. Revised and Enlarged, with four hundred and sixty-one Illustrations. W. T. Keener Company, Chicago, Ill., 1894.

This, the third edition of Dr. Talbot's work on deformities has been so increased in number of pages, illustrations, and valuable material that it is practically another book added to the former editions.

These were carefully reviewed as they appeared, and nothing can be added to strengthen the conviction that the work of Dr. Talbot stands, upon the subject upon which it treats, as the most important contribution yet given on the osseous deformities of the head and face.

While the conclusions may not be always accepted, the careful arrangement of facts must prove of immense value in determining disputed questions, and will form a basis for the solution of many intricate problems and racial defects.

The general trend of the author's work may be gathered from the following quotation: "The law of the 'survival of the fittest' affects not only the entire organism, but also the parts themselves. Some one part attains undue development. Such a product of degeneracy once obtained might, under proper conditions, remain, while the rest of the body returned to the normal type. The frequency with which these stigmata of degeneracy are found in otherwise sound systems indicates that the law of atavism tends to eradicate as well as to cause them. Given these deformities in a subject of normal mental health, they simply indicate that a more or less remote ancestor had been subject to the influence of the factors producing degeneracy, but that in the main the offspring were regaining the normal standard.

"The lesson to be drawn from these stigmata is the hygienic one that, given the tendency to degeneracy shown in these deformities, the progress of that person under the factors named will tend more towards disease than that of the person in whom they are absent. The frequency of these deformities should hence have early created the suspicion that they were of constitutional and not local origin, and should have once more taught the old, old lesson in medicine,—that a knowledge of man's ancestry is of great value in treatment."

Space will not permit a thorough analysis of this work; indeed, any review would be unsatisfactory, as it belongs to a rare class of books that requires careful and prolonged study, and this it must receive in order that it may be comprehended.

Chapter XXXIX. consists of a series of thirty-six plates illustrating "the upper jaws of boys from an orphan asylum, ranging from six to twelve years of age," also thirty-six pages with outline "antero-posterior and lateral illustrations of the vault."

